# Prospective Evaluation of the Cardiovascular Effects of BRAF and MEK Inhibitors in Patients With Melanoma

**DOI:** 10.1016/j.jaccao.2025.08.006

**Published:** 2025-10-10

**Authors:** Claire Glen, Stephen J.H. Dobbin, Kenneth Mangion, Alasdair Henderson, Katriona Brooksbank, Caroline J. Coats, Frederick H. Epstein, Peter Kellman, Elaine Butler, Thomas R. Jeffry Evans, Rob Jones, John McClure, Giles Roditi, Yun Yi Tan, Ashita Waterston, Paul Welsh, Mark C. Petrie, Ninian N. Lang

**Affiliations:** a School of Cardiovascular and Metabolic Health, University of Glasgow, Glasgow, Scotland, United Kingdom; b Queen Elizabeth University Hospital, NHS Greater Glasgow and Clyde, Glasgow, Scotland, United Kingdom; c School of Engineering and Applied Science, University of Virginia, Charlottesville, Virginia, USA; d National Heart Lung and Blood Institute, National Institutes of Health, Bethesda, Maryland, USA; e University of Glasgow, Glasgow, Scotland, United Kingdom; f Beatson West of Scotland Cancer Centre, NHS Greater Glasgow and Clyde, Glasgow, Scotland, United Kingdom; g Institute of Cancer Sciences, University of Glasgow, Glasgow, Scotland, United Kingdom

**Keywords:** biomarkers, cancer therapy–related cardiac dysfunction, cardiac magnetic resonance, echocardiography, melanoma, risk prediction, targeted therapy

## Abstract

**Background:**

Rapidly accelerated fibrosarcoma B-type (BRAF) and MEK inhibitors have revolutionized outcomes for patients with BRAF-mutated melanoma. However, they are associated with cardiovascular adverse effects. The real-world incidence and risk factors for these effects are poorly described.

**Objectives:**

The aim of this study was to characterize the incidence and risk factors for BRAF inhibitor– and MEK inhibitor–associated hypertension and cancer therapy–related cardiac dysfunction (CTRCD) in a real-world setting.

**Methods:**

A prospective, longitudinal, cohort study was undertaken among patients with melanoma treated with BRAF and MEK inhibitors in a regional cancer network (March 2021 to March 2023). Baseline cardiotoxicity risk stratification was assessed using the European Society of Cardiology cardio-oncology guideline–recommended tool. Comprehensive cardiovascular assessment was performed at baseline and at 4, 12, and 24 weeks, including home and clinic blood pressure, echocardiography, stress perfusion cardiovascular magnetic resonance imaging and blood biomarkers. CTRCD was defined using International Cardio-Oncology Society definitions.

**Results:**

A total of 61 participants were enrolled. Twenty-eight participants (45.9%) developed hypertension and 45.9% developed CTRCD: 24 (85.7%) mild, 3 (10.7%) moderate, and 1 (3.6%) severe. All moderate or severe CTRCD was evident by 4 weeks and at least partially reversible. No patient at low baseline risk developed moderate or severe CTRCD. Patients with CTRCD had higher median baseline N-terminal pro–B-type natriuretic peptide compared with those without (109 pg/mL [Q1-Q3: 51-380 pg/mL] vs 54 pg/mL [Q1-Q3: 29-149 pg/mL]; *P* = 0.047). There were no robust associations between hypertension nor cardiovascular magnetic resonance imaging–derived myocardial or perfusion characteristics and incident CTRCD.

**Conclusions:**

BRAF inhibitor– and MEK inhibitor–associated hypertension and CTRCD are common. The present results reinforce the utility of baseline cardiotoxicity risk stratification, including assessment of N-terminal pro–B-type natriuretic peptide. Future guidelines should consider recommending early surveillance echocardiography for higher risk patients.

The discovery of targeted therapies against BRAF and MEK can be considered among the most substantial advances in anticancer treatment in recent years. BRAF inhibitors (BRAFi) include dabrafenib, vemurafenib, and encorafenib, while MEK inhibitors (MEKi) include trametinib, binimetinib and cobimetinib. These drugs act upon the MAPK pathway, which plays a key role in cell proliferation, differentiation, and apoptosis to achieve cancer disease control. However, the MAPK pathway also plays a central role in cardiac and vascular cell signaling, and its pharmacologic manipulation can be associated with unwanted cardiovascular effects.^1^ Recognized BRAFi- and MEKi-associated cardiovascular adverse effects (CVAEs) include hypertension, left ventricular systolic dysfunction (LVSD), QT interval prolongation, atrial arrhythmia, and venous thromboembolism.^2^^,^^3^ The mechanisms underlying these cardiovascular toxicities are incompletely understood. However, disruption of the MAPK pathway impairs physiological myocardial protective mechanisms. In the context of exposure to BRAFi or MEKi, myocardial resilience to a further stressor such as hypertension or ischemia may be lowered.^3^

BRAFi and MEKi were initially approved for the treatment of advanced unresectable BRAF-mutated melanoma, for which treatment options were limited and prognosis poor. However, their use has broadened, and they are now used in the adjuvant setting, with curative intent, for stage III BRAF-mutated melanoma^4^ and are also approved for use in other cancers, including non–small cell lung cancer^5^ and colorectal cancer.^6^ These growing indications further highlight the importance of understanding the mechanisms, incidence, and timing of associated CVAEs to inform risk stratification, surveillance, and treatment. The majority of data relating to BRAFi- and MEKi-associated CVAEs are from clinical trials.^7^, ^8^, ^9^, ^10^, ^11^ However, there are substantial challenges with the interpretation of these data. Indeed, clinical trial reporting and definitions of adverse effects are heterogeneous,^12^ and they generally exclude or under-represent patients with pre-existing cardiovascular disease and risk factors.^13^ Despite the widespread use BRAFi and MEKi, no prior prospective study has specifically assessed their cardiovascular effects.

The European Society of Cardiology (ESC) cardio-oncology guidelines^14^ provide recommendations to risk-stratify patients prior to treatment with targeted therapies using the Heart Failure Association/International Cardio-Oncology Society cardiotoxicity risk tool.^15^ Other recommendations include the periodic monitoring of blood pressure (BP) and electrocardiography in all patients and echocardiographic assessment of left ventricular ejection fraction (LVEF) depending upon baseline risk category. However, these recommendations are not based on high-quality evidence, and the cardiotoxicity risk tool has not undergone robust validation in this patient group.

We performed a prospective investigation of the cardiovascular effects of BRAFi and MEKi in patients with melanoma. We examined risk factors for CVAEs and categorized participants using the ESC-recommended baseline cardiotoxicity tool. In addition to comprehensive serial home BP monitoring, echocardiography, and electrocardiography, we incorporated deeper assessment including blood-based biomarkers and stress perfusion cardiovascular magnetic resonance imaging (CMR) to provide novel, granular insights to inform clinical practice and guideline development (Central Illustration).

**Central Illustration:**
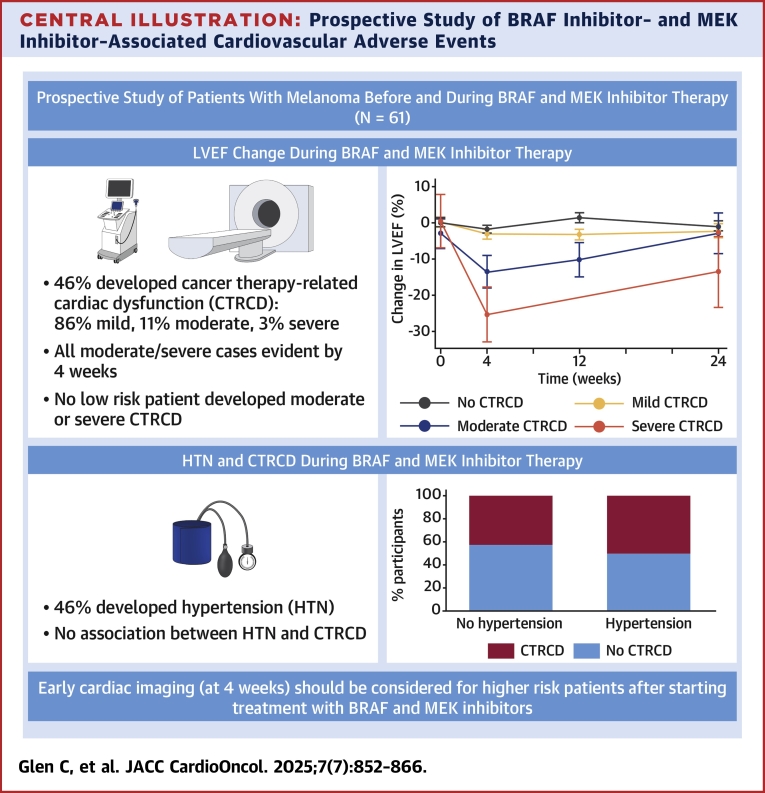
Prospective Study of BRAF Inhibitor– and MEK Inhibitor–Associated Cardiovascular Adverse Events A total of 61 patients with melanoma treated with BRAF and MEK inhibitors underwent detailed cardiovascular assessments for the first 6 months of therapy. Key findings are shown. Cancer therapy–related cardiac dysfunction (CTRCD) was common, occurring in 46% of participants, but in the majority this was mild. Treatment-associated hypertension also occurred in 46% of patients, but there was no clear association between hypertension and incident CTRCD. HTN = hypertension; LVEF = left ventricular ejection fraction.

## Methods

### Study population

We conducted a prospective, observational, cohort study of participants treated in a regional cancer hospital network (West of Scotland Cancer Network, National Health Service, United Kingdom; March 2021 to May 2023). Patients with melanoma considered to be suitable for therapy with BRAFi and MEKi, ≥18 years of age, with Eastern Cooperative Oncology Group performance status 0 or 1, and with predicted survival ≥6 months were invited to participate. The only exclusion criterion was the inability to provide informed consent. All participants were invited to take part in an optional stress perfusion CMR substudy with additional exclusion criteria of standard contraindications to CMR, estimated glomerular filtration rate < 20 mL/min/1.73 m^2^, persistent or permanent atrial fibrillation (AF) (because of the potential for image quality degradation), and second- or third-degree atrioventricular block. This study was approved by the West of Scotland Research Ethics Service (20/WS/0166), and all participants provided informed consent.

### Data collection

**Figure 1:**
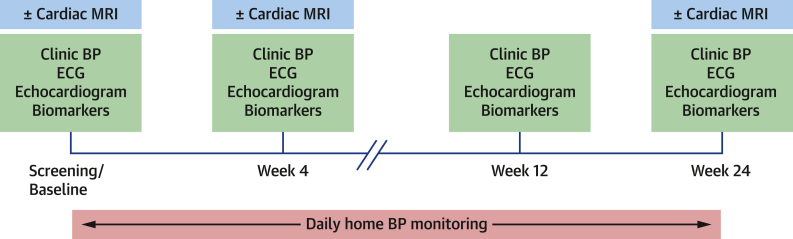
Schedule of Study Assessments Diagram of study schedule and assessments carried out at each visit. BP = blood pressure; ECG = electrocardiography; MRI = magnetic resonance imaging.

The schedule of study assessments is shown in Figure 1. All participants attended baseline assessments prior to the commencement of BRAFi and MEKi. Demographic and clinical data were collected through history, clinical examination, and review of electronic medical records. The Heart Failure Association/International Cardio-Oncology Society baseline risk assessment tool^15^ was used to categorize patients as low, medium, high, or very high risk for incident cardiotoxicity (Supplemental Table 1). Follow-up visits were performed 4, 12, and 24 weeks after starting treatment. Blood samples were collected for high-sensitivity troponin T (hsTnT) and N-terminal pro–B-type natriuretic peptide (NT-proBNP) measurement. These were centrifuged and serum was frozen at −80°C and subsequently batch-analyzed using Elecsys Troponin T hs and Elecsys proBNP II (Roche Diagnostics International) assays, respectively. A threshold of “abnormal” NT-proBNP (>125 pg/mL) was used, in keeping with ESC cardio-oncology guidelines.^14^ The abnormal hsTnT threshold (>14 pg/mL) was determined by the 99th percentile for the Elecsys Troponin T hs assay, without use of sex-specific cutoffs, also in keeping with ESC cardio-oncology guideline recommendations.^14^ Troponin and NT-proBNP were measured at the end of the study, and those results were not available for routine clinical care.

### BP

Clinic BP was measured prior to commencement of BRAFi or MEKi therapy. Participants were provided with a clinically validated, automatic oscillometric BP monitor (M2, Omron) and advised to monitor BP at the same time each day, taking 2 measurements, at least 1 minute apart, and to record the average of these. Seven-day average BP readings were calculated. At each study visit, participants were asked to describe their home BP measurement technique to ensure optimal practice, and BP diaries were assessed to ensure protocol adherence. BRAFi- or MEKi-associated hypertension was defined as a 7-day average home BP value of ≥135 mm Hg systolic or ≥85 mm Hg diastolic, where at least 3 days of measurements were recorded in a 7-day period and/or the initiation or escalation of existing antihypertensive therapy in line with ESC and International Society of Hypertension guidelines.^16^^,^^17^ In the event of uncontrolled baseline BP greater than this threshold, the definition was met by an increase in systolic BP or diastolic BP of ≥20 mm Hg from baseline, and/or the escalation of existing antihypertensive therapy as per the National Cancer Institute Common Terminology Criteria for Adverse Events version 5.0.^18^

### Echocardiography

Echocardiography was performed at baseline and at 4, 12, and 24 weeks after starting BRAFi and MEKi. Structured assessment was performed using British Society of Echocardiography data set standards (Supplemental Table 2).^19^ Images were acquired by C.G. and 2 senior cardiac physiologists on a Siemens Acuson SC2000 (Siemens Healthineers) or a GE Vivid E95 (GE Healthcare) machine. All images were reported by a single reader blinded to participant characteristics (C.G.) on a vendor-agnostic platform (EchoPAC version 204, revision 57.6, GE Healthcare) to reduce bias. LVEF was calculated using the modified Simpson’s biplane method.^19^ Global longitudinal strain (GLS) was measured by tracing endocardial borders in 3 apical views (2-, 3-, and 4-chamber views). By tracing the endocardial border in end-systole, myocardial speckles were automatically tracked in subsequent frames and manually corrected if inadequate tracking was identified. GLS analysis was not performed if there was impaired regional tracking in >2 contiguous myocardial segments.

In accordance with the International Cardio-Oncology Society CTRCD definitions, BRAFi- or MEKi-associated mild asymptomatic CTRCD was defined as a reduction in GLS of >15% relative to baseline, with LVEF remaining at ≥50% and/or new increases in cardiac biomarkers. New increases in cardiac biomarkers were defined as cardiac troponin > 99th percentile or NT-proBNP ≥ 125 pg/mL or a new significant rise from baseline beyond the biological and analytical variation of the assay used as per International Cardio-Oncology Society definitions. For patients with biomarker values greater than these thresholds at baseline, a subsequent 20% increase was considered relevant. Moderate asymptomatic CTRCD was defined as a reduction in LVEF to <50% plus either a ≥10% LVEF reduction from baseline or a smaller change in LVEF with a concomitant decrease in GLS by >15% relative to baseline or a new increase in cardiac biomarkers. Severe asymptomatic CTRCD was defined as any new reduction in LVEF to <40%.^12^ Recovery was defined as improvement in LVEF to at least 50%.

### CMR and analyses

Stress perfusion CMR was performed in a subgroup of participants. Scans were performed at 3 time points over the 6-month follow-up study period (baseline, 4 weeks, and 24 weeks) using a 3.0-T magnetic resonance imaging scanner (Magnetom Prisma, Siemens Healthineers) with a standard phased-array cardiac surface coil. The protocol included cine imaging, T1 and T2 parametric mapping, extracellular volume (ECV), displacement encoding with stimulated echoes, perfusion, and late gadolinium enhancement sequences. Perfusion imaging was performed at rest and under stress conditions. The stress agent, adenosine, was prepared in 0.9% sodium chloride to a volume of 1 mg/mL and administered intravenously at 140 to 210 μg/kg/min to achieve an adequate stress response. Hyperemia was confirmed by a hemodynamic response (defined as a heart rate increase >10 beats/min and a systolic BP decrease >10 mm Hg and/or the onset of typical symptoms [flushing, chest tightness, dyspnea]). Study analyses were performed by a single reader (C.G.) blinded to subject identity, clinical data and time point of the scan. Further details, including CMR protocol (Supplemental Table 3), are available in the Supplemental Appendix.

### Statistical analysis

All data are presented as mean ± SD or median (Q1-Q3) according to distribution. Categorical variables are presented as numbers and percentages with 95% CIs. Baseline characteristics were summarized according to outcome of CTRCD. Student’s *t*-test, the Mann-Whitney *U* test, or the chi-square test was used to determine the associations of baseline characteristics with the development of CTRCD. The chi-square test was used to assess the association between Heart Failure Association/International Cardio-Oncology Society cardiotoxicity baseline risk category and the development of CTRCD. Repeated-measures mixed-effects models were used to examine the change in LVEF, relative change in GLS from baseline, and log-transformed mean biomarker concentrations over time according to CTRCD status. Results are presented as the least squares mean with 95% CI at each time point. Models were adjusted for baseline of the outcome variable (LVEF, GLS, or biomarker), visit, and the interaction of CTRCD status and visit using a random intercept and slope per participant and an unstructured covariance. Models were not adjusted for covariates. A normal distribution was used, and assumptions were tested by examining the plot of residuals and fitted values. Missing data were considered to be missing at random, and imputation was not performed. Because of the exploratory nature of the study, no adjustment was made for multiple testing, and there was no a priori formal power calculation.

All analyses were performed using Stata version 17 software (StataCorp). Statistical significance was defined as 2-tailed *P* value <0.05 for all tests.

## Results

### Study population

**Figure 2:**
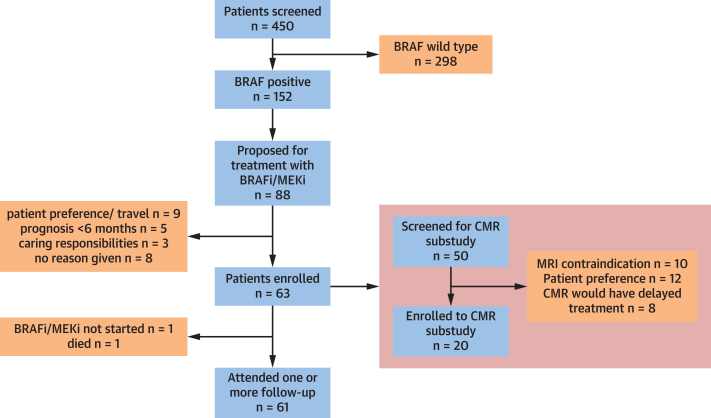
Consolidated Standards of Reporting Trials Diagram Flow diagram of participant progress throughout study follow-up. BRAFi = BRAF inhibitor; CMR = cardiovascular magnetic resonance imaging; MEKi = MEK inhibitor.

A total of 63 participants were recruited (Figure 2). One participant was commenced on immunotherapy rather than BRAFi or MEKi, and another participant had a rapid decline in health status following the initiation of BRAFi and MEKi and died prior to week 4 follow-up. These patients were excluded from analysis, resulting in 61 participants included at baseline. Fifty-seven participants (93.4%) completed the full 24-week follow-up. Fifty participants (82.0%) received the combination of dabrafenib plus trametinib, and 11 (18.0%) received encorafenib plus binimetinib. Fifty-five participants (90.2%) were initiated on full-dose BRAFi and MEKi therapy. Forty-eight participants (78.7%) were treated in the adjuvant setting, and 12 participants (19.7%) had metastatic disease.

Baseline characteristics of the overall study population are shown in Table 1. The mean age was 59 ± 15 years, and 60.7% were male. Sixteen (26.2%) had histories of hypertension, 1 (1.6%) had a history of LVSD, 3 (4.9%) had prior myocardial infarctions, 5 (8.2%) had AF, and 8 (13.1%) had diabetes.

**Table 1:** Baseline Characteristics (N = 61)

Age, y	59 ± 15
Male	37 (60.7)
BMI, kg/m^2^	28.4 ± 6.2
White race	61 (100)
Smoking history	
Current	6 (9.8)
Former	13 (21.3)
Never	42 (68.9)
Cancer stage	
3	50 (82.0)
4	11 (18.0)
AJCC 8th edition melanoma staging	
3a	4 (6.6)
3b	20 (32.8)
3c	23 (37.7)
3d	3 (4.9)
4	11 (18.0)
Previous cancer treatment	
Immune checkpoint inhibitors	7 (11.5)
BRAFi/MEKi	3 (4.9)
Radiotherapy	2 (3.3)
Surgery	54 (88.5)
Treatment indication	
Adjuvant	48 (78.7)
Palliative	13 (21.3)
BRAFi/MEKi type	
Dabrafenib plus trametinib	50 (82.0)
Encorafenib plus binimetinib	11 (18.0)
Medical history	
LVSD	1 (1.6)
Hypertension	16 (26.2)
Myocardial infarction	3 (4.9)
Previous PCI	1 (1.6)
Previous CABG	2 (3.3)
Diabetes	8 (13.1)
Atrial fibrillation	5 (8.2)
Hypercholesterolemia	5 (8.2)
Stroke	1 (1.6)
CV medications	
Beta-blockers	9 (14.8)
ACE inhibitors	12 (19.7)
ARBs	2 (3.3)
Aspirin	7 (11.5)
Anticoagulation	6 (9.8)
Non-rate-limiting CCBs	12 (19.7)
Statins	16 (26.2)
Loop diuretic agents	3 (4.9)
Thiazide diuretic agents	3 (4.9)
MRAs	1 (1.6)

Values are mean ± SD or n (%). Percentages may not total 100 because of rounding.

ACE = angiotensin-converting enzyme; AJCC = American Joint Committee on Cancer; ARB = angiotensin receptor blocker; BMI = body mass index; BRAFi = BRAF inhibitor; CABG = coronary artery bypass grafting; CCB = calcium-channel blocker; LVSD = left ventricular systolic dysfunction; MEKi = MEK inhibitor; MRA = mineralocorticoid receptor antagonist; PCI = percutaneous coronary intervention.

### BRAFi- and MEKi-associated CTRCD

Twenty-eight participants (45.9%) developed CTRCD following initiation of BRAFi and MEKi treatment, of whom 24 (85.7%) were classified with mild, 3 (10.7%) with moderate, and 1 (3.6%) with severe asymptomatic CTRCD. No participant developed symptomatic heart failure. Baseline characteristics according to the development of CTRCD by severity are shown in Table 2. A history of AF was associated with the development of any severity of CTRCD; all 5 participants with AF developed CTRCD (*P* = 0.011), 4 classified as mild CTRCD and 1 as severe CTRCD. There was no difference in baseline LVEF or left ventricular dimensions in those who developed any severity of CTRCD vs those who did not (Table 3), and those who developed CTRCD had lower baseline GLS compared with those who did not (−18.0% ± 2.5% vs −16.8% ± 1.7%; *P* = 0.029).

**Table 2:** Baseline Characteristics of Participants With and Without CTRCD

	All Participants (N = 61)	No CTRCD (n = 33)	Mild CTRCD (n = 24)	Moderate CTRCD (n = 3)	Severe CTRCD (n = 1)
Age, y	59 ± 15	57 ± 15	60 ± 17	65 ± 10	76
Male	37 (60.6)	18 (54.5)	17 (70.8)	2 (66.7)	0 (0)
BMI, kg/m^2^	28.4 ± 6.2	28.8 ± 6.8	28.1 ± 5.2	25.3 ± 7.6	31.4
Smoking history					
Current	6 (9.8)	3 (9.1)	2 (8.3)	1 (33.3)	0 (0)
Former	13 (21.3)	9 (27.3)	3 (12.5)	1 (33.3)	0 (0)
Never	42 (68.9)	21 (63.6)	19 (79.2)	1 (33.3)	1 (100)
AJCC 8th edition melanoma staging					
3a	4 (6.6)	2 (6.1)	2 (8.3)	0 (0)	0 (0)
3b	20 (32.8)	13 (39.4)	6 (25.0)	1 (33.3)	0 (0)
3c	23 (37.7)	13 (39.4)	8 (33.3)	1 (33.3)	1 (100)
3d	3 (4.9)	0 (0)	2 (8.3)	1 (33.3)	0 (0)
4	11 (18.0)	5 (15.2)	6 (25.0)	0 (0)	0 (0)
BRAFi/MEKi type					
Dabrafenib plus trametinib	50 (82.0)	28 (84.8)	18 (75.0)	3 (100)	1 (100)
Encorafenib plus binimetinib	11 (18.0)	5 (15.2)	6 (25.0)	0 (0)	0 (0)
Full-dose BRAFi at initiation	57 (93.4)	32 (97.0)	21 (87.5)	3 (100)	1 (100)
Full-dose MEKi at initiation	55 (90.2)	31 (93.9)	20 (83.3)	3 (100)	1 (100)
Treatment indication					
Adjuvant	48 (78.7)	27 (81.8)	17 (70.8)	3 (100)	1 (100)
Palliative	13 (21.3)	6 (18.2)	7 (29.2)	0 (0)	0 (0)
Prior immune checkpoint inhibitor therapy	7 (11.5)	2 (6.1)	5 (20.8)	0 (0)	0 (0)
Medical history					
LVSD	1 (1.6)	0 (0)	0 (0)	1 (33.3)	0 (0)
HTN	16 (26.2)	6 (18)	8 (33)	1 (33)	1 (100)
HTN (uncontrolled)	12 (19.7)	5 (15)	7 (29)	0 (0)	0 (0)
MI	3 (4.9)	1 (3)	1 (4)	1 (33)	
Diabetes	8 (13.1)	5 (15)	3 (13)	0 (0)	0 (0)
Atrial fibrillation	5 (8.2)	0 (0)	4 (17)	0 (0)	1 (100)
Hypercholesterolemia	5 (8.2)	4 (12)	1 (4)	0 (0)	0 (0)
Stroke	1 (1.6)	0 (0)	1 (4)	0 (0)	0 (0)
Peripheral vascular disease	2 (3.3)	1 (3)	1 (4)	0 (0)	0 (0)
Valvular heart disease	3 (4.9)	0 (0)	3 (13)	0 (0)	0 (0)
Baseline CV medications					
Beta-blockers	9 (14.8)	1 (3.0)	5 (20.8)	2 (66.7)	1 (100)
ACE inhibitors	12 (19.7)	3 (9.1)	6 (25.-)	2 (66.7)	1 (100)
ARBs	2 (3.3)	1 (3.0)	1 (4.2)	0 (0)	0 (0)
Aspirin	7 (11.5)	2 (6.1)	4 (16.7)	1 (33.3)	0 (0)
Anticoagulation	6 (9.8)	2 (6.1)	3 (12.5)	0 (0)	1 (100)
Dihydropyridine CCBs	12 (19.7)	4 (12.1)	7 (29.2)	0 (0)	1 (100)
Rate-limiting CCBs	1 (1.6)	0 (0)	1 (4.2)	0 (0)	0 (0)
Statins	16 (26.2)	7 (21.2)	7 (29.2)	2 (66.7)	0 (0)
Loop diuretic agents	3 (4.9)	1 (3.0)	1 (4.2)	1 (33.3)	0 (0)
Thiazide diuretic agents	3 (4.9)	2 (6.1)	1 (4.2)	0 (0)	0 (0)
MRAs	1 (1.6)	0 (0)	0 (0)	1 (33.3)	0 (0)

Values are presented as mean ± SD or n (%). Percentages may not total 100 because of rounding.

HTN = hypertension; MI = myocardial infarction; other abbreviations as in Table 1.

**Table 3:** Baseline Echocardiographic Findings in Patients With and Without CTRCD

	All Participants (N = 61)	No CTRCD (n = 33)	Any CTRCD (n = 28)	*P* Value
LV structure and function				
LVIDd, mm	43.4 ± 4.5	42.6 ± 4.6	44.4 ± 4.2	0.11
LVIDs, mm	28.9 ± 4.0	29.3 ± 4.3	28.4 ± 3.6	0.39
IVSd, mm	8.9 ± 1.7	8.8 ± 1.7	9.0 ± 1.8	0.75
LVEF, %	61.9 ± 5.1	62.0 ± 5.8	61.7 ± 4.1	0.85
GLS, %	−17.4 ± 2.2	−16.8 ± 1.7	−18.0 ± 2.5	0.029
LV diastolic function				
E, m/s	0.7 (0.6-0.8)	0.6 (0.5-0.8)	0.7 (0.6-0.8)	0.13
A, m/s	0.7 ± 0.1	0.7 ± 0.1	0.6 ± 0.1	0.042
E/A ratio	1.0 (0.8-1.2)	0.8 (0.8-1.1)	1.1 (0.9-1.3)	0.047
E′ lateral	11.5 ± 3.3	11.4 ± 3.3	11.6 ± 3.4	0.87
E′ septal	9.2 ± 2.6	9.0 ± 2.4	9.5 ± 2.8	0.50
E/E′ ratio (average)	6.8 (5.3-7.5)	6.5 (5.3-7.4)	6.8 (4.9-7.5)	0.64
LA area, cm^2^	15.0 (12.0-19.0)	15.0 (13.0-18.0)	15.5 (11.5-20.5)	0.78
TR Vmax, m/s	2.2 (2.0-2.7)	2.2 (2.1-2.3)	2.1 (2.0-2.8)	0.95
RV structure and function				
Mid-RV diameter, mm	27.1 ± 4.1	26.7 ± 4.1	27.7 ± 4.1	0.31
RV s′, cm/s	13.3 ± 2.7	13.1 ± 2.7	13.6 ± 2.7	0.48
TAPSE, cm	22.7 ± 3.6	22.8 ± 3.5	22.5 ± 3.9	0.71

Values are mean ± SD, or median (Q1-Q3).

CTRCD = cancer therapy–related cardiac dysfunction; GLS = global longitudinal strain; IVSd = interventricular septal diameter at end-diastole; LA = left atrial; LVEF = left ventricular ejection fraction; LVIDd = left ventricular internal diameter in diastole; LVIDs = left ventricular internal diameter in systole; RV = right ventricular; TAPSE = tricuspid annular plane systolic excursion; TR Vmax = maximal tricuspid regurgitation velocity.

When classified using the ESC-recommended baseline risk score, 29 patients (47.5%) were considered at low risk for the development of cardiotoxicity. Of these, 11 (37.9%) developed mild CTRCD, but none developed moderate or severe CTRCD. Overall, 4 participants (6.6%) developed moderate or severe CTRCD. Of these, 1 participant (1.6%) was considered at medium risk, 2 participants (3.3%) at high risk, and 1 participant (1.6%) at very high risk for the development of cardiotoxicity. Thus, participants who developed moderate or severe CTRCD were all considered to be at least medium risk at baseline (*P* = 0.004) (Table 4).

**Table 4:** Association of Baseline Risk Score and Development of CTRCD

HFA-ICOS Risk Score	All	No CTRCD	Mild CTRCD	Moderate CTRCD	Severe CTRCD
Low	29 (47.5)	18 (54.5)	11 (45.8)	0	0
Medium	13 (21.3)	6 (18.2)	6 (25.0)	1 (33.3)	0
High	18 (39.5)	9 (27.3)	7 (29.2)	1 (33.3)	1 (100)
Very high	1 (1.6)	0	0	1 (33.3)	0

Values are n (%). Percentages may not total 100 because of rounding.

CTRCD = cancer therapy–related cardiac dysfunction; HFA = Heart Failure Association; ICOS = International Cardio-Oncology Society.

**Figure 3:**
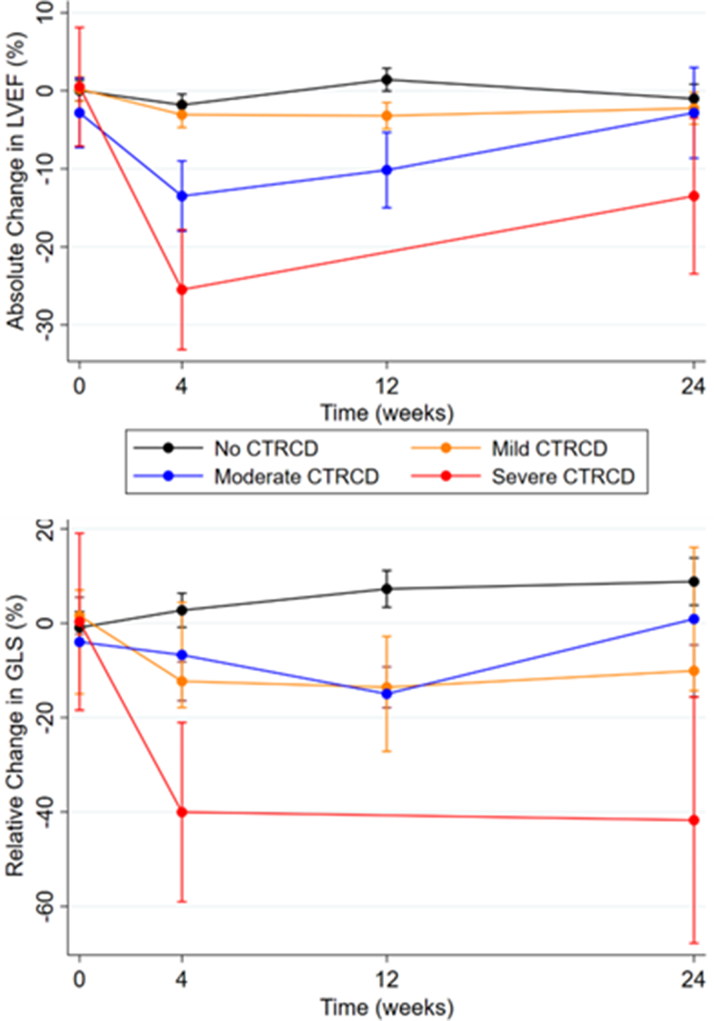
LVEF and GLS During Treatment With BRAF and MEK Inhibitors The black line represents no cancer therapy–related cardiac dysfunction (CTRCD), the blue line represents mild CTRCD, the orange line represents moderate CTRCD, and the red line represents severe CTRCD. Circles indicate the least squares mean, and whiskers indicate 95% CIs. GLS = global longitudinal strain; LVEF = left ventricular ejection fraction.

Moreover, 24 participants (39.3%) developed mild CTRCD, and this first occurred at 4 weeks in 10 participants (41.7%), at 12 weeks in 10 (41.7%), and 24 weeks in 4 (16.7%). Following the visit with decline in GLS, there was recovery to baseline in 10 participants (41.7%) and persistent >15% reduction in GLS in 14 (58.3%). No participants were commenced on potentially cardioprotective medications or had BRAFi or MEKi dose alterations on the basis of mild CTRCD alone. Of those with mild CTRCD, none progressed to moderate or severe CTRCD. Change in GLS during follow-up is shown in Figure 3.

Four participants (6.6%) developed moderate or severe CTRCD (3 moderate, 1 severe), and this occurred at 4 weeks in all participants. Changes in LVEF during follow-up are shown in Figure 3. All participants with CTRCD were receiving treatment in the adjuvant setting and had at least temporary interruption of both BRAFi and MEKi therapy as per local oncologic practice at the time of this study. Decisions regarding interruption of therapy were at the discretion of the oncology team. In 2 cases of moderate CTRCD, LVEF recovered to >50% on repeat echocardiography, BRAFi and MEKi treatment was reintroduced, and LVEF remained normal thereafter. In the third case, BRAFi and MEKi was permanently discontinued at 4 weeks because of noncardiovascular toxicities. An angiotensin-converting enzyme inhibitor was commenced when CTRCD was identified and LVEF subsequently recovered to 53% at 24 weeks. In the fourth case, the participant developed severe asymptomatic CTRCD and had a decline in LVEF to 37% (baseline LVEF 63%). BRAFi and MEKi therapy was permanently discontinued, and following treatment with a beta-blocker, an angiotensin-converting enzyme inhibitor, a mineralocorticoid receptor antagonist, and a sodium-glucose cotransporter 2 inhibitor, LVEF was 49% at 24 weeks.

### BRAFi- and MEKi-associated hypertension

**Figure 4:**
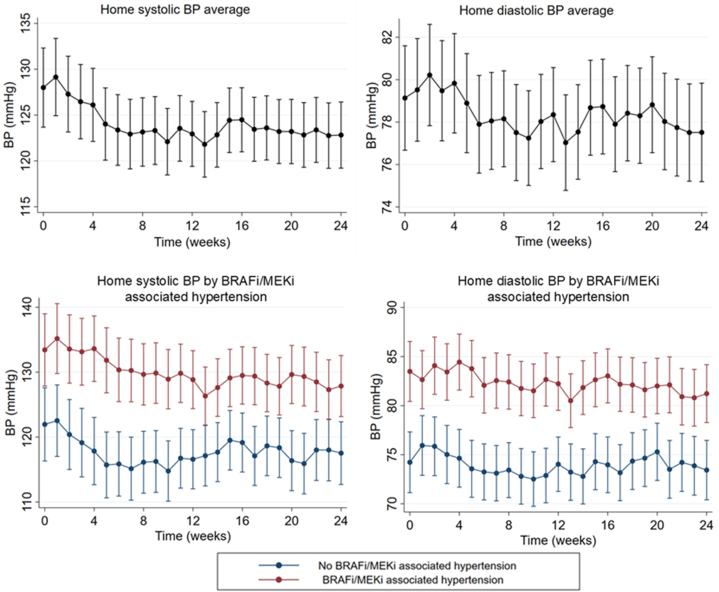
Weekly Average BP Across Follow-Up in all Patients and in Those Who Developed Treatment-Associated Hypertension The black line represents all patients, the red line represents those who developed treatment-associated hypertension, and the blue line represents those who did not develop treatment-associated hypertension. Circles indicate the least squares mean, and whiskers indicate 95% CIs. Abbreviations as in Figures 1 and 2.

**Figure 5:**
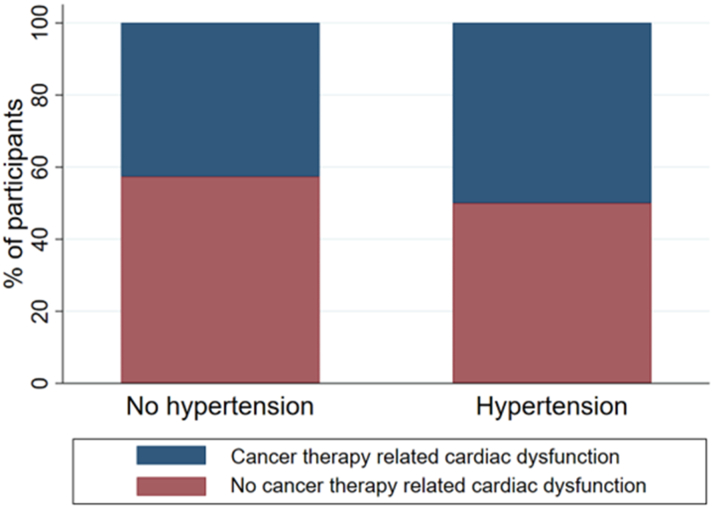
Association of BRAF Inhibitor– and MEK Inhibitor–Associated Hypertension and Cancer Therapy–Related Cardiac Dysfunction Bar chart demonstrating no strong association between incident BRAF inhibitor– and MEK inhibitor–associated hypertension and incident cancer therapy–related cardiac dysfunction.

Baseline home systolic BP was 128 ± 16 mm Hg and diastolic BP was 79 ± 11 mm Hg. Following treatment, 28 participants (45.9%) met the criteria for BRAFi- or MEKi-associated hypertension, of whom 12 (42.9%) had isolated diastolic hypertension. No participants had modification of BRAFi or MEKi dose because of uncontrolled hypertension. However, 16 participants had dose reduction or discontinuation because of other toxicities. Seven-day average BP across follow-up in all patients and in those who developed treatment-associated hypertension is shown in Figure 4. Treatment of hypertension was at the discretion of the oncology team. Eleven participants received antihypertensive treatment (calcium-channel blockers in 36.4%, angiotensin-converting enzyme inhibitors in 45.5%, and other medications in 18.2%). The presence of BRAFi- or MEKi-associated hypertension was not associated with the development of CTRCD (Figure 5).

### Cardiac biomarkers

At baseline, the median NT-proBNP concentration was 80 pg/mL (Q1-Q3: 33-209 pg/mL), and it was elevated in 24 participants (39.3%). Those who developed any severity of CTRCD had higher NT-proBNP at baseline compared with those without CTRCD (109 pg/mL [Q1-Q3: 51-380 pg/mL] vs 54 pg/mL [Q1-Q3: 29-149 pg/mL]; *P* = 0.047).

At baseline, median hsTnT was 5.8 pg/mL (Q1-Q3: 4.1-10.9 pg/mL), and it was elevated in 7 participants (11.5%). Those who developed any severity of CTRCD had numerically higher baseline hsTnT compared with those with no CTRCD, but this was not statistically significant (7.7 pg/mL [Q1-Q3: 4.9-13.1 pg/mL] vs 4.8 pg/mL [Q1-Q3: 4.0-9.0 pg/mL]; *P* = 0.067).

**Figure 6:**
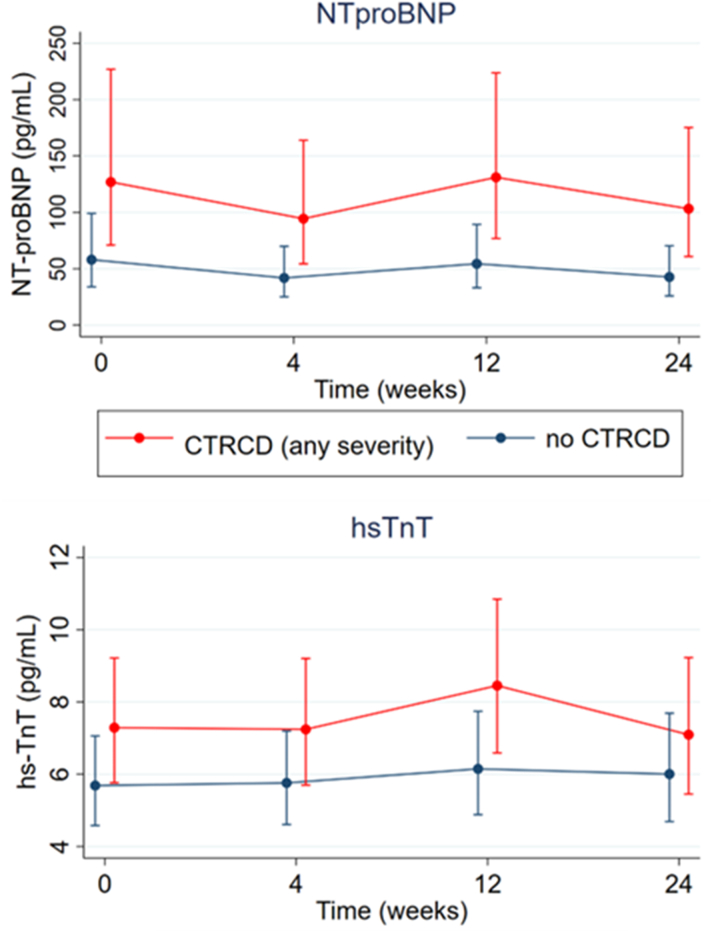
NT-proBNP and hsTnT During Treatment With BRAF and MEK Inhibitors The blue line represents no cancer therapy–related cardiac dysfunction (CTRCD), and the red line represents CTRCD (any severity). Circles indicate the least squares mean, and whiskers indicate 95% CIs. hsTnT = high-sensitivity troponin T; NT-proBNP = N-terminal pro–B-type natriuretic peptide.

Participants who developed any severity of CTRCD continued to have higher levels of hsTnT and NT-proBNP during follow-up compared with those with no CTRCD, but there was no change over time (Figure 6). All participants who met criteria for CTRCD did so on the basis of echocardiographic criteria, and the incorporation of the “new rise in cardiac biomarkers” criterion did not identify any further cases.

### CMR substudy

Twenty participants from the study population were enrolled in the CMR substudy. Apart from sex, there were no differences in baseline characteristics in those who took part in the substudy compared with those who did not (Supplemental Table 4). Mean LVEF and GLS during follow-up are shown in Supplemental Figure 1. The pattern and magnitude of changes in LVEF and GLS by CMR were similar to those measured using echocardiography. Five participants (25.0%) had declines in LVEF to <50%, but only 1 participant (5.0%) had a coexisting change in LVEF ≥10% from baseline to fulfill criteria for the diagnosis of moderate CTRCD. This participant was also diagnosed with moderate CTRCD on echocardiography. Three (15.0%) had elevated T1 at baseline, and 5 (25.0%) went on to develop elevated T1 during follow-up (reference range: 1,107-1,234 ms), but there was no association with elevated baseline T1 and the development of CTRCD (*P* = 0.47). One participant (5.0%) developed an elevated T2 during follow-up (reference range: 35-44 ms), and no participants had abnormal ECV during follow-up. Myocardial perfusion was normal in all participants at baseline and during follow-up. T1, T2, ECV, and myocardial perfusion reserve during follow-up are shown in Supplemental Figure 2.

### Other CVAEs and noncardiovascular adverse events

No participant developed a prolongation of corrected QT interval or new cardiac arrhythmia during treatment with BRAFi or MEKi. There were no significant changes in electrocardiographic parameters throughout therapy (Supplemental Figure 3). Three participants died during the 24-week follow-up period, 2 of cancer progression and 1 of probable pulmonary embolism. Twenty-three participants (37.7%) had dose reductions and 3 (4.9%) permanently discontinued treatment during the follow-up period for noncardiovascular toxicities.

## Discussion

To the best of our knowledge, this is the first study to make a specific prospective assessment of the cardiovascular effects of BRAFi and MEKi. Incorporating contemporary definitions and the ESC-recommended baseline risk stratification tool, we have demonstrated that BRAFi- and MEKi-associated CTRCD is common but was predominantly mild in severity and asymptomatic. Importantly, in those who developed moderate or severe CTRCD, this occurred early during the course of BRAFi and MEKi treatment and was first evident at 4 weeks. Those who developed moderate or worse CTRCD all met criteria for at least medium risk for CTRCD when assessed at baseline using the risk stratification tool. BRAFi- and MEKi-associated hypertension was also common, occurring in 45.9% of participants, but was not clearly associated with the development of CTRCD. Elevated NT-proBNP at baseline was associated with higher risk for the development of CTRCD.

This study builds upon our previous retrospective analysis of routine clinical cardiovascular assessments in patients with melanoma treated with BRAFi and MEKi. In that study, more patients had incomplete follow-up, and the granularity of data was more limited. The present prospective study presents a much more comprehensive, protocol-driven assessment of cardiovascular toxicities including cardiac biomarkers, home BP monitoring, and serial echocardiography, and missing data were substantially reduced. By including a serial stress perfusion CMR substudy, we have provided deeper cardiac characterization.

The observed 45.9% incidence of any severity of CTRCD is higher than the 27.0% incidence we observed in our prior retrospective analysis.^20^ However, the higher incidence observed in the present prospective study is driven largely by a greater incidence of mild CTRCD, and the 6.6% incidence of moderate and severe CTRCD is similar to that observed in the retrospective study.^20^ The higher overall incidence of CTRCD in the present study is likely to at least partly reflect the robust prospective analyses and completeness of follow-up. Indeed, at 24 weeks, only 4 participants (6.6%) were not assessed, in comparison with the absence of analyzable data in about 50% of patients at the same time point in the retrospective study.^20^ Although clinical trial assessments have varied, incident LVSD has been reported in 2% to 12% of participants.^7^, ^8^, ^9^, ^10^, ^11^^,^^21^

Although there was a high incidence of mild CTRCD in this study, none of these participants subsequently developed moderate or severe CTRCD. The ESC cardio-oncology guidelines recommend the measurement of GLS in all patients with cancer undergoing echocardiography.^14^ By definition, LVEF is normal in those with mild CTRCD, and our results have shown that over the 24 weeks of follow-up, no participant with mild CTRCD went on to develop moderate or severe CTRCD. There are no specific recommendations for treatment of BRAFi- or MEKi-associated mild CTRCD. However, our results further reinforce that, similar to recommendations for mild CTRCD associated with more comprehensively studied anticancer therapies such as trastuzumab, BRAFi and MEKi treatment should not be modified on the basis of mild CTRCD alone.

The ESC cardio-oncology guidelines^14^ advise that the need for echocardiographic surveillance for patients treated with BRAFi and MEKi should be determined by the baseline cardiotoxicity risk score. For those in high or very high risk categories, it is recommended that echocardiography every 4 months be considered. In our study, 47.5% of participants were considered to be at low risk for incident cardiotoxicity according to the risk stratification tool. Although 37.9% of these developed mild CTRCD, none developed moderate or severe CTRCD, and our results therefore support this use of the baseline risk stratification tool to determine those for whom routine echocardiographic surveillance is of less relevance. However, our findings suggest that in those deemed to be at higher risk (potentially also including those in the medium baseline risk group), earlier echocardiographic follow-up at 4 weeks may be more appropriate. Indeed, all incident moderate and severe CTRCD cases were evident by 4 weeks, with no new cases identified at later assessments. In the context of the observed reversibility of CTRCD and scope for resumption of BRAFi and MEKi treatment, the early identification of CTRCD is of high relevance to allow the optimal balance between cardiovascular health and ongoing cancer therapy. We did not observe any cases of symptomatic heart failure. The early identification of asymptomatic CTRCD may have mitigated this but this remains speculative, and we also acknowledge that the study was not adequately powered to make further assertions on this.

Cardiovascular biomarkers including troponin and NT-proBNP are included in the baseline cardiotoxicity risk score calculator. At baseline, 39.3% of participants had elevated NT-proBNP (>125 pg/mL). Although higher baseline NT-proBNP values were associated with the development of CTRCD, NT-proBNP did not change over time, suggesting that the primary utility of NT-proBNP is in baseline risk stratification, rather than for ongoing cardiovascular monitoring. For patients with CTRCD, the longitudinal assessment of NT-proBNP may serve as an early marker of incipient heart failure, but given that no patients in this study developed symptomatic CTRCD, this is not addressable here. Furthermore, given that NT-proBNP levels may themselves be affected by the presence of cancer, particularly in more advanced malignancy, knowledge of baseline values remains important for later comparisons.^22^^,^^23^

Hypertension is the most common adverse effect reported in clinical trials of BRAFi and MEKi therapies, with a reported incidence of 6% to 26%.^2^^,^^3^ The incidence in our cohort was higher than previously reported, occurring in 45.9% of participants. This is likely to reflect the robust use of home BP monitoring, and we believe that this finding further reinforces the clinical relevance of home BP measurement in this high-risk group. In clinical practice, it may be acceptable to reduce the frequency of monitoring from that described here, and in some patients it may be deemed inappropriate to undertake monitoring at all, such as in those with advanced metastatic disease. Underlying pathophysiologic mechanisms for the development of BRAFi- and MEKi-associated hypertension and LVSD have not been extensively studied. It has previously been hypothesized that LVSD may be a result of BRAFi and MEKi plus a second hit of hypertension or ischemia,^3^ but in our cohort, there was no relation between BRAFi- or MEKi-associated hypertension and change in LVEF or GLS in the treatment period studied. It is possible that because of robust BP monitoring in this study, any potential association between BP and cardiac dysfunction may have been diluted. This may be particularly relevant to GLS, which is an afterload-dependent metric. Given the lack of robust association between BP and CTRCD, direct myocardial effects of BRAFi and MEKi remain important considerations. The longer term effects of uncontrolled BP related to BRAFi and MEKi therapy requires further study and are particularly relevant to those treated in the adjuvant setting.

Our stress perfusion CMR substudy included myocardial tissue characterization with T1 and T2 mapping and assessment of ECV in an endeavor to investigate underlying mechanisms, and potential baseline and incident myocardial risk markers, of BRAFi- and MEKi-associated CTRCD. Although 5 participants (25.0%) had decreases in LVEF to <50%, only 1 (5.0%) had a coexisting change in LVEF ≥10% from baseline representative of moderate CTRCD, which is comparable with the incidence of moderate CTRCD of 4.9% in the main cohort. Although a serial, multiparametric CMR stress perfusion study of this size is notable in the assessment of cardiotoxicity of anticancer therapy, the low incidence of moderate CTRCD in this group limits the conclusions that can be drawn. However, our previous study incorporating serial stress perfusion CMR in patients receiving VEGF inhibitors revealed substantial changes in myocardial perfusion and tissue characteristics following treatment with that drug class despite a much smaller sample size.^24^ This is of relevance because it may previously have been tempting to draw parallels between VEGF inhibitor–associated and BRAFi- or MEKi-associated hypertension and CTRCD. Our findings highlight that these 2 different classes of targeted anticancer therapy should not be considered interchangeably.

### Study limitations

Strengths of this novel, real-world study include its prospective design, providing an accurate depiction of the longitudinal cardiovascular effects of BRAFi and MEKi, as well as the ability to compare against pretreatment baseline values. The incorporation of comprehensive cardiovascular assessments, including echocardiography, batch-analyzed biomarkers, and CMR, enhances the detection and characterization of cardiac dysfunction. The use of contemporary definitions of CTRCD as recommended by the International Cardio-Oncology Society and ESC guidelines ensures relevance and comparability with other studies. Additionally, we used the ESC-recommended baseline cardiotoxicity risk stratification tool, which aids in assessing the utility of this tool in the study population.

However, although this is the only prospective study to focus on cardiovascular effects of BRAFi and MEKi, the sample size limited statistical power. The follow-up period was relatively short (6 months), so we cannot draw any conclusions on the later effects of BRAFi- and MEKi-associated adverse effects. Long-term outcome evaluation, including the assessment of progression to symptomatic heart failure, is an important area for future work. All participants were White, and our results might not be applicable to other racial groups. Larger, multicenter assessments including registries from geographically and racially diverse populations and centers will be important for future study. Mixed models were not adjusted for covariates. Although several variables could have been considered, in a sample of this size it is likely that adjustment for these would have resulted in an overfitted model. Longitudinal results presented were not corrected for time varying exposure, and we did not correct for multiple pairwise comparisons of baseline characteristics, and therefore these results require validation in larger cohort studies.

## Conclusions

CTRCD and hypertension are common CVAEs of BRAFi and MEKi. More than one-half of the participants did not develop any CTRCD, and although about 40% developed mild CTRCD in this real-world patient group, this did not become more severe during 24 weeks of follow-up. Therefore, most patients tolerated treatment without unacceptable cardiovascular effects. However, the risk for moderate and severe CTRCD remains relevant and was evident as early as 4 weeks after starting BRAFi and MEKi in those at risk. Our findings support the utility of baseline cardiotoxicity risk stratification, including the measurement of NT-proBNP. Serial BP assessment should be performed routinely, and our findings highlight the value of home measurement. We provide data to suggest that future cardio-oncology guidelines should consider recommending early echocardiographic assessment (at 4 weeks) for all patients treated with BRAFi or MEKi, except those deemed to be in the lowest baseline risk group. Future studies might specifically evaluate the appropriateness of lower intensity cardiac imaging surveillance in patients deemed to be at low baseline risk, and potentially also those who have reassuring findings at 4 weeks. These efforts to risk-stratify and perform appropriate cardiovascular surveillance in patients treated with BRAFi or MEKi should allow patients to receive optimal anticancer therapy while minimizing the risk for associated cardiovascular morbidity.

Perspectives **COMPETENCY IN MEDICAL KNOWLEDGE:** BRAFi- and MEKi-associated CTRCD is common, predominantly mild in severity and asymptomatic. Moderate or worse CTRCD was evident by 4 weeks after therapy initiation and occurred in those considered at medium risk for cardiotoxicity. Therefore, early follow-up at 4 weeks should be considered in anyone at greater than low risk. Baseline NT-proBNP and home BP monitoring should be considered in patients treated with BRAFi and MEKi.

**TRANSLATIONAL OUTLOOK:** Future research is needed to assess the long-term cardiovascular effects of BRAFi and MEKi and to validate our results, particularly with regard to risk stratification in a larger cohort.

## Funding Support and Author Disclosures

Drs Lang and Glen are supported by a restricted grant from Roche Diagnostics. Drs Lang and Petrie are supported by a British Heart Foundation Centre of Research Excellence Grant (RE/18/6/34217). Dr Lang has received research grants from Roche Diagnostics, AstraZeneca, and Boehringer Ingelheim; and has received consulting and speaking fees from Roche Diagnostics, MyoKardia, Pharmacosmos, Akero Therapeutics, CV6 Therapeutics, Jazz Pharma, and Novartis, all outside the submitted work. Dr Welsh has received grant income from Roche Diagnostics, AstraZeneca, Boehringer Ingelheim, and Novartis; and has received speaking fees from Novo Nordisk and Raisio, all outside the submitted work. Dr Roditi has received speaking honoraria from GE Healthcare and Bracco; and is a consulting, without remuneration, for Canon Medical. Dr Tan has received personal fees from Novartis. Dr Evans has received honoraria for consultancies (payable to the employing institution) from Ascelia, AstraZeneca, Bayer, Bicycle Therapeutics, Bristol Myers Squibb, Celgene, Eisai, Karus Therapeutics, Medivir, Merck Sharpe & Dohme, Otsuka, Roche, and Seagen; has received honoraria for speaking fees (payable to the employing institution) from AstraZeneca, Ascelia, Bayer, Bristol Myers Squibb, Celgene, Eisai, Nucana, Merck Sharpe & Dohme, Roche, Medivir, and United Medical; and has received funding from Cancer Research UK, Chief Scientist’s Office Scotland, and the Medical Research Council (payable to the employing institution). Dr Jones has received personal fees from Astellas, AstraZeneca, Bristol Myers Squibb, Bayer, Exelixis, Janssen, Ipsen, Merck Serono, Merck Sharpe & Dohme, Novartis, Pfizer, Roche, Sanofi Genzyme, and EUSA; and has received research grant funding from Astellas, AstraZeneca, Bayer, Exelixis, and Roche. Dr Petrie has received research funding from Boehringer Ingelheim, Roche, SQ Innovations, AstraZeneca, Novartis, Novo Nordisk, Medtronic, Boston Scientific, Pharmacosmos; is a consultant and trial committee member for Abbott Laboratories, Akero, Applied Therapeutics, Amgen, AnaCardio, Biosensors, Boehringer Ingelheim, Corteria, Novartis, AstraZeneca, Novo Nordisk, Abbvie, Bayer, Horizon Therapeutics, Foundry, Takeda, Cardiorentis, Pharmacosmos, Siemens, Eli Lilly, Vifor, New Amsterdam, Moderna, Teikoku, LIB Therapeutics, 3R Lifesciences, Reprieve, FIRE 1, Corvia, and Regeneron. All other authors have reported that they have no relationships relevant to the contents of this paper to disclose.
