# Supplementary material for: Prospective Evaluation of the Cardiovascular Effects of BRAF and MEK Inhibitors in Patients With Melanoma
Source: JACC CardioOncol. 2025 Oct 10;7(7):852–66. doi: 10.1016/j.jaccao.2025.08.006 (PMC12805401; doi:10.1016/j.jaccao.2025.08.006)
Supplement: Supplementary Material [file mmc1.docx]

**Prospective evaluation of the cardiovascular effects of BRAF and MEK inhibitors in patients with melanoma:**

***A study incorporating risk scores, cardiac biomarkers and cardiovascular magnetic resonance imaging***

**Supplemental Material**

Supplemental figure 1 ……………………………………………………. page 2

Supplemental figure 2 ……………………………………………………. page 3

Supplemental figure 3 ……………………………………………………. page 4

Supplemental table 1 ……………………………………………………. page 5

Supplemental table 2 ………………………………………………………. page 7

Supplemental table 3 …………………………………………………….. page 8

Supplemental table 4………………………………………………………….. page 9

Supplemental appendix ……………………………………………………. page 11

References……………………………………………………………………. page 15


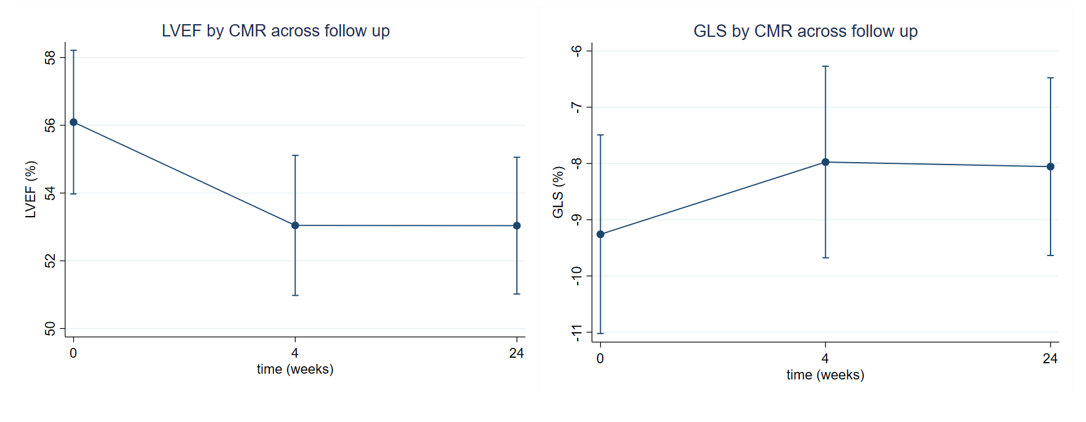


Supplemental Figure 1. LVEF and GLS as measured by CMR across follow up.


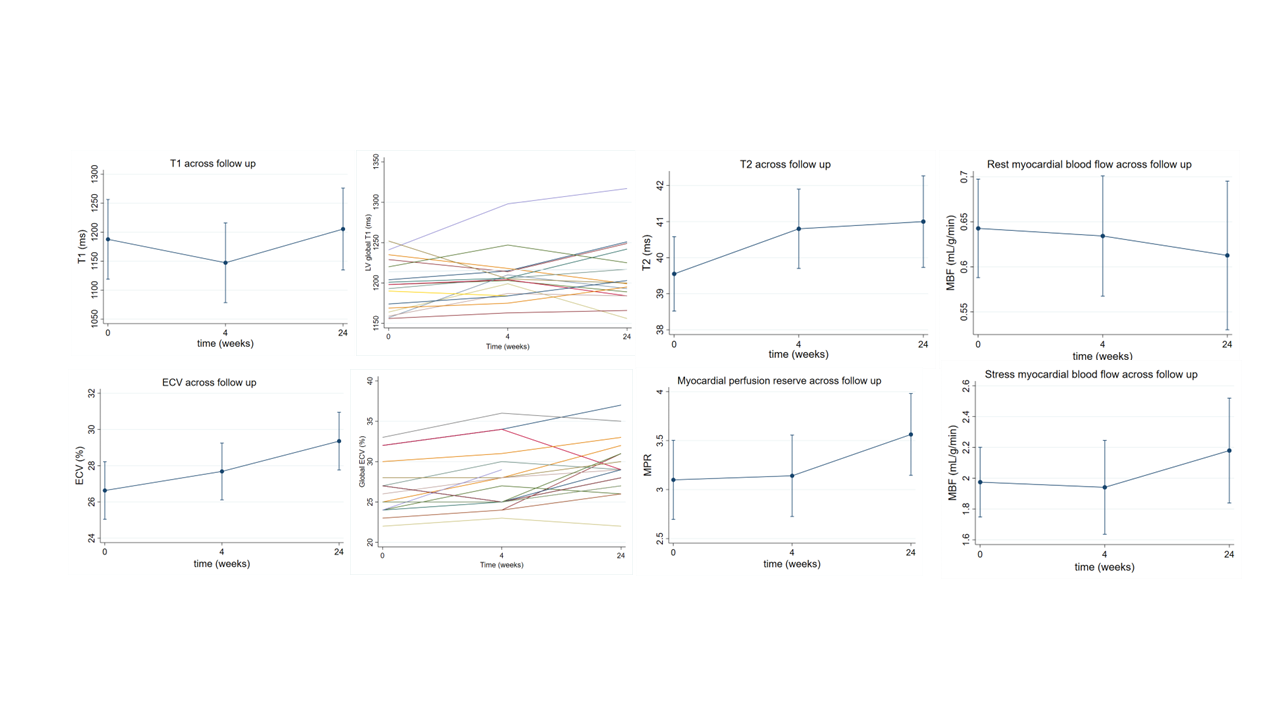
Supplemental Figure 2. T1, T2, ECV, MPR, stress and rest myocardial blood flow across follow up as measured by CMR, mean values and individual participants.


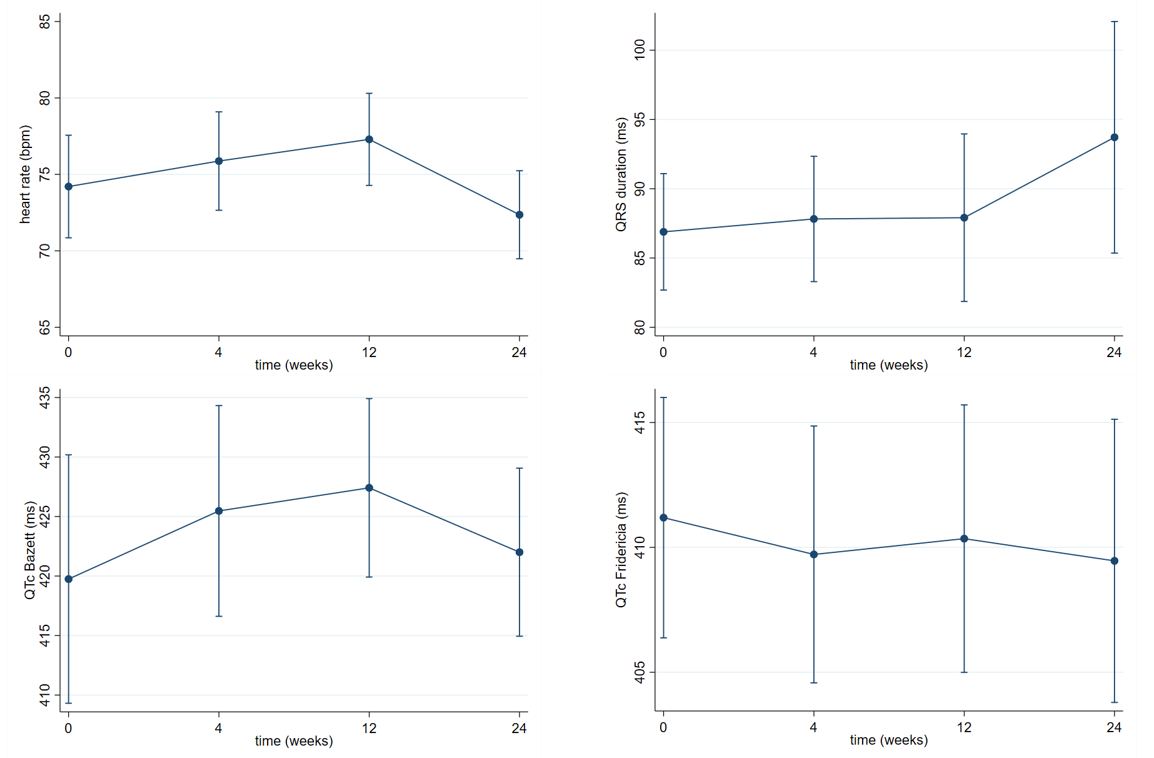


Supplemental Figure 3. ECG parameters across follow up

| **Supplemental Table 1. HFA-ICOS baseline risk assessment tool** | |
| --- | --- |
| **Risk Factor** | **Score** |
| ***Previous cardiovascular disease*** |  |
| Heart failure or cardiomyopathy | Very high |
| Myocardial infarction or CABG | High |
| Stable angina | High |
| Severe valvular heart disease | High |
| Borderline LVEF 50-54% | Medium^2^ |
| Arrhythmia^a^ | Medium^1^ |
| ***Cardiac biomarkers (when available)*** |  |
| Elevated baseline troponin^b^ | Medium^2^ |
| Elevated baseline BNP or NT-proBNP^b^ | Medium^2^ |
| ***Demographic and cardiovascular risk factors*** |  |
| Age ≥ 65 years | Medium^1^ |
| Hypertension^c^ | Medium^2^ |
| Diabetes Mellitus^d^ | Medium^1^ |
| Chronic kidney disease^e^ | Medium^1^ |
| ***Previous cardiotoxic cancer treatment*** |  |
| Prior anthracycline exposure^f^ | High |
| Prior radiotherapy to left chest or mediastinum | Medium^2^ |
| ***Lifestyle risk factors*** |  |
| Current smoker or significant smoking history | Medium^1^ |
| Obesity (BMI > 30kg/m^2^) | Medium^1^ |
| BMI, body mass index; BNP, brain natriuretic peptide; CABG, coronary artery bypass graft; LVEF, left ventricular ejection fraction; NT-proBNP, N-terminal pro-brain natriuretic peptide  Low risk = no risk factor or one Medium^1^ risk factor  Medium risk = medium risk factors with a total of 2-4 points  High risk = medium risk factors with a total of ≥ 5 points OR any high risk factor  Very high risk = any very high risk factor  ^a^ Atrial fibrillation, atrial flutter, ventricular tachycardia, or ventricular fibrillation.  ^b^ Elevated above the upper limit of normal for local laboratory reference range.  ^c^ Systolic blood pressure >140mmHg or diastolic blood pressure >90 mmHg, or on treatment.  ^d^ Glycated haemoglobin >7.0% or >53 mmol/mol, or on treatment.  ^e^ Estimated glomerular filtration rate <60 mL/min/1.73m2.  ^f^ Previous malignancy | |


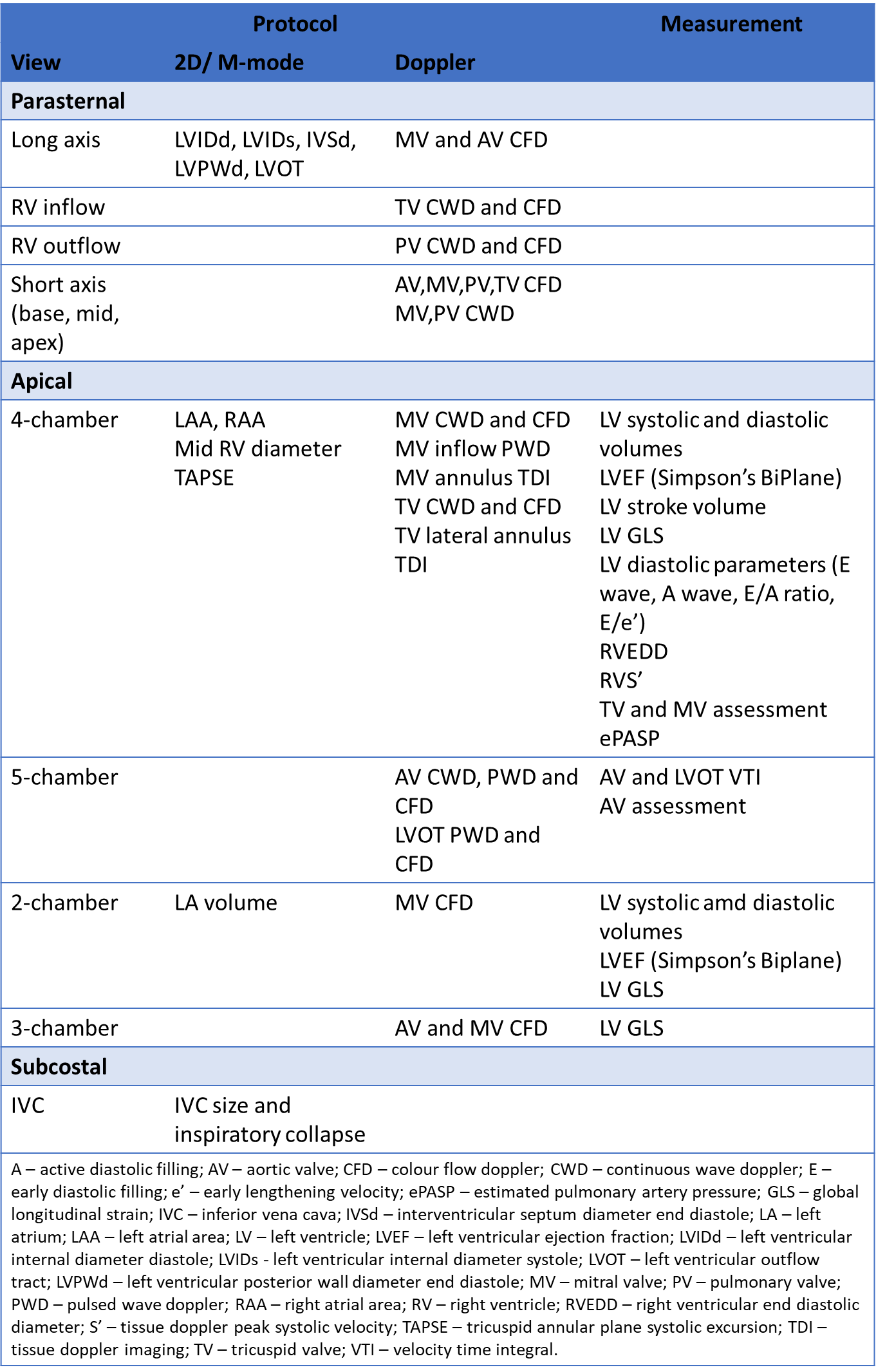


Supplemental Table 2. Echocardiography protocol

| **Supplemental Table 3. Cardiac magnetic resonance protocol** | | | | | |  |  |
| --- | --- | --- | --- | --- | --- | --- | --- |
| **Parameter** | **Cine** | **T1** | **T2** | **DENSE** | **Stress perfusion** | **Rest perfusion** | **Late gadolinium** |
| **Orientation** | HLA, VLA LVOT, SA stack | HLA, Transverse AIF*, SA (basal,mid,apex) | SA (basal,mid,apex) | HLA,VLA,LVOT, SA (basal,mid,apex) | SA (basal,mid,apex), transverse AIF* | SA (basal,mid,apex), transverse AIF* | HLA, VLA LVOT, SA stack |
| **Sequence** | bSSFP | MOLLI | SSFP | * | SR-TurboFLASH | SR-TurboFLASH | PSIR TurboFLASH |
| **Breath-hold** | Breath-hold | Breath-hold | Breath-hold | Breath-hold | Gentle breathing | Gentle breathing | Breath-hold |
| **TR (ms)** | 41.4 | Pre: 264 Post: 341 | 207 | 27 | 144 | 144 | 671.68 |
| **TE (ms)** | 1.51 | 1.01 | 1.32 | 1.26 | 0.98 | 0.98 | 1.05 |
| **TI (ms)** | n/a | Pre: 180 Post: 260 | n/a |  | 90 | 90 | 250-350 |
| **Flip angle** | 50^o^ | 35^o^ | 12^o^ | 15^o^ | 10^o^ | 10^o^ | 14^o^ |
| **FOV(mm*mm)** | 340x286 | 360x306 | 360x270 | 360x200 | 360x270 | 360x270 | 350x262 |
| **Matrix (mm)** | 216x256 | 218x256 | 154x192 |  | 144x192 | 144x192 | 192x111 |
| **Slice thickness (mm)** | 7 | 8 | 8 | 8 | 8 | 8 | 8 |
| **Slice gap (mm)** | 3 | n/a | n/a | n/a | n/a | n/a | 3 |
| **Acceleration** | n/a | GRAPPA-2 | GRAPPA-2 | * | GRAPPA-2 | GRAPPA-2 | GRAPPA-2 |
| **Bandwith (Hz/px)** | 962 | 1085 | 1184 | 1207 | 1184 | 1184 | 1221 |
| *Transverse aortic plane at level of the main pulmonary artery Abbreviations: AIF, arterial input function; bSSFP, balanced steady-state free precession; FOV, field of view; GRAPPA, generalised autocalibrating partial parallel acquisition; HLA, horizontal long axis; LVOT, left ventricular outflow tract; MOLLI, modified Lock-Locker inversion recovery; n/a, not applicable; SA, short axis; SR-Turbo-FLASH, saturation recovery turbo fast low-angle shot; TE, echo time; TI, inversion time; TR, repetition time; VLA, vertical long axis | | | | | | | |

| **Supplemental Table 4. Baseline characteristics by participants who underwent cardiac MRI and those who did not** | | | | |
| --- | --- | --- | --- | --- |
|  | **Total** | **No Cardiac MRI** | **Cardiac MRI** | p-value |
|  | N=61 | N=41 | N=20 |  |
| **Age (years)** | 59 ±15 | 61 ± 15 | 54 ± 14 | 0.066 |
| **Male sex, no. (%)** | 37 (60.7) | 20 (48.9) | 17 (85.0) | 0.006 |
| **BMI (kg/m^2^)** | 28.4 ± 6.2 | 28.8 ± 6.9 | 27.5 ± 4.3 | 0.43 |
| **Smoking History, no.(%)** |  |  |  | 0.16 |
| Current | 6 (9.8) | 5 (12.2) | 1 (5.0) |  |
| Ex-smoker | 13 (21.3) | 6 (14.6) | 7 (35.0) |  |
| Never smoked | 42 (68.9) | 30 (73.2) | 12 (60.0) |  |
| **Cancer stage** |  |  |  | 0.25 |
| Stage 3 | 50 (82.0) | 32 (78.0) | 18 (90.0) |  |
| Stage 4 | 11 (18.0) | 9 (22.0) | 2 (10.0) |  |
| **AJCC 8^th^ Edition Melanoma Staging** |  |  |  | 0.26 |
| Stage 3a | 4 (6.6) | 1 ( 2.4) | 3 (15.0) |  |
| Stage 3b | 20 (32.8) | 15 (36.9) | 5 (25.0) |  |
| Stage 3c | 23 (37.7) | 14 (34.1) | 9 (45.0) |  |
| Stage 3d | 3 (4.9) | 2 (4.9) | 1 (5.0) |  |
| Stage 4 | 11 (18.0) | 9 (22.0) | 2 (10.0) |  |
| **HFA-ICOS risk score** |  |  |  | 0.51 |
| Low | 29 (47.5) | 19 (46.3) | 10 (50.0) |  |
| Medium | 13 (21.3) | 9 (22.0) | 4 (20.0) |  |
| High | 18 (39.5) | 13 (31.7) | 5 (25.0) |  |
| Very high | 1 (1.6) | 0 (0) | 1 (5.0) |  |
| **BRAF/MEK type** |  |  |  | 0.67 |
| Dabrafenib plus trametinib | 50 (82.0) | 33 (80.5) | 17 (85.0) |  |
| Encorafenib plus binimetinib | 11 (18.0) | 8 (19.5) | 3 (15.0) |  |
| **Treatment indication** |  |  |  | 0.40 |
| Adjuvant | 48 (78.7) | 31 (75.6) | 17 (85.0) |  |
| Palliative | 13 (21.3) | 10 (24.4) | 3 (15.0) |  |
| **Past Medical History** |  |  |  |  |
| LVSD | 1 (1.6) | 0 (0) | 1 (5.0) | 0.15 |
| HTN | 16 (26.2) | 10 (24.3) | 6 (30.0) | 0.64 |
| MI | 3 (4.9) | 1 (2.4) | 2 (10.0) | 0.25 |
| Diabetes | 8 (13.1) | 3 (7.3) | 5 (25.0) | 0.055 |
| Atrial fibrillation | 5 (8.2) | 5 (12.2) | 0 (0) | 0.10 |
| Hypercholesterolaemia | 5 (8.2) | 2 (4.9) | 3 (15.0) | 0.18 |
| Cerebrovascular disease | 1 (1.62) | 1 (2.4) | 0 (0) | 0.48 |
| Peripheral Vascular disease | 2 (3.3) | 1 (2.4) | 1 (5.0) | 0.60 |
| Valvular heart disease | 3 (4.9) | 1 (2.4) | 1 (5.0) | 0.99 |
| **Baseline CV medications** |  |  |  |  |
| Beta-blocker | 9 (14.8) | 6 (14.6) | 3 (15.0) | 0.97 |
| ACE inhibitor | 12 (19.7) | 5 (12.2) | 7 (35.0) | 0.035 |
| ARB | 2 (3.3) | 2 (4.8) | 0 (0) | 0.32 |
| Sacubitril/valsartan | 0 (0) | 0 (0) | 0 (0) |  |
| Aspirin | 7 (11.5) | 3 (7.3) | 4 (20.0) | 0.14 |
| Anticoagulation | 6 (9.8) | 6 (14.6) | 0 (0) | 0.072 |
| Non rate limiting CCB | 12 (19.7) | 7 (11.5) | 5 (25.0) | 0.46 |
| Rate limiting CCB | 1 (1.6) | 0 (0) | 1 (5.0) | 0.15 |
| Statin | 16 (26.2) | 8 (19.5) | 8 (40.0) | 0.088 |
| Nitrate | 0 (0) | 0 (0) | 0 (0) |  |
| Loop diuretic | 3 (4.9) | 2 (4.9) | 1 (5.0) | 0.98 |
| Thiazide diuretic | 3 (4.9) | 2 (4.9) | 1 (5.0) | 0.98 |
| MRA | 1 (1.6) | 0 (0) | 1 (5.0) | 0.15 |
| Abbreviations: ACE, angiotensin converting enzyme; ARB, angiotensin receptor blocker; BMI, body mass index; CABG, coronary artery bypass grafting; CCB, calcium channel blocker; HTN, hypertension; LVSD, left ventricular systolic dysfunction; MI, myocardial infarction; MRA, mineralocorticoid receptor antagonist; PCI, percutaneous coronary intervention.  Data are presented as mean ± SD for continuous measures, and n (%) for categorical measures. | | | | |

**Supplemental Appendix**

**Cardiac Magnetic Resonance**

Stress perfusion cardiac magnetic resonance imaging was performed in a sub-group of participants. The CMR sub-study opened on 1/8/21 therefore the first 13 participants in the study were not screened for enrolment in the sub-study. All subsequent participants recruited were screened for eligibility for enrolment in the sub-study.

Balanced steady-state free precession (SSFP) sequences were used to acquire ventricular cine imaging in three long axis planes (2, 3 and 4 chamber), followed by a short axis stack from the apex to the atrio-ventricular ring, each with 30 phases, for assessment of cardiac function. Three left ventricular short axis (basal, mid and apical) and one orthogonal long axis longitudinal relaxation time (T1, spin–lattice relaxation time constant in milliseconds) motion-corrected, optimized, modified Look-Locker inversion recovery sequences^1,2^ were acquired. A short axis stack of T2 prep SSFP^3^ (T2, spin–spin relaxation time constant in milliseconds) maps and orthogonal long axis views were acquired, followed by an automated exponential fit for each pixel after respiratory motion correction. Global myocardial extracellular volume (ECV) fraction was analysed by manually contouring LV endocardial and epicardial myocardium and LV blood pool in a single short axis mid-LV slice in both pre- and post-contrast T1 maps. Global extracellular volume fraction was then calculated from pre- (native) and post-contrast myocardial and blood pool T1 values, together with a hematocrit taken on the same day^4^.

Displacement encoding with stimulated echoes (DENSE) sequences^5^ were acquired in three short-axis (basal, mid-ventricular, apical) and three long-axis (horizontal long axis, vertical long axis and left ventricular outflow tract) to assess longitudinal and circumferential strain. Segmentation of the LV myocardium was performed semi-automatically after endo- and epicardial contours were drawn, using the anterior right ventricular insertion point as reference. Spatio-temporal phase unwrapping was then carried out on the LV myocardium pixels, and displacement vectors were calculated^6,7^ . Lagrangian strain was computed from these displacements and then projected into radial, circumferential (or longitudinal in long axis acquisitions) directions relative to the left ventricular centre of mass. Data was exported as text files.

Late gadolinium enhancement images, including three long axis acquisitions and a short axis stack, were acquired 10–15minutes after intravenous injection of 0.15mmol kg^-1^ of gadolinium using segmented phase-sensitive inversion recovery sequences.

Perfusion imaging was performed at rest and under stress conditions. Stress agent adenosine was prepared in 0.9% sodium chloride to a volume of 1mg/mL and administered intravenously at a rate of 140 to 210 micrograms/kg/min to achieve an adequate haemodynamic stress response. Hyperaemia was confirmed by a haemodynamic response defined as heart rate increase >10 beats per minute and systolic blood pressure decrease >10 mmHg and/or the onset of typical symptoms (flushing, chest tightness, dyspnoea). A total dose of 0.15 mmol/kg of gadolinium-based contrast (Gadovist) was administered (0.05mmol/kg bolus for first-pass stress perfusion, 0.05 mmol/kg blous for rest perfusion and 0.05 mmol/kg top-up bolus for LGE imaging).

The perfusion sequence used has been previously described^8^ . In summary, the sequence used a dual-sequence approach with separate pulse sequences for the arterial input function (AIF) and myocardial tissue. Images were acquired over 90 heart beats with a bolus of 0.05mmol/kg gadolinium-based contrast administered at 4mL/s followed by a 20mL flush. The LV blood pool signal was automatically segmented from optimised low-resolution proton density-weighted images acquired in parallel with higher spatial resolution saturation recovery images and used to calculate the AIF. Myocardial perfusion was calculated using an automated blood tissue exchange model, correcting for T2* losses and non-linearity of saturation recovery. Pixel-wise perfusion maps were automatically generated in-line and available to view on the scanner. Maps were corrected for motion and surface-coil intensity prior to generation. All scans were analysed for the presence of splenic switch off as a measure of adequate hyperaemia. Visual comparison of splenic contrast enhancement on the short axis slice in which the spleen was best seen was performed and switch off was graded as either present (lower splenic enhancement at stress compared to rest) or absent (no obvious difference in splenic enhancement between stress and rest).

After deidentification, MRI scans were reviewed and reported by a single image analyst (CG). Ventricular volumes, mass, ejection fraction and motion-corrected T1 and T2 sequences were analysed using dedicated software (cvi42 software (version 5.10, Circle Cardiovascular)). DENSE data was analysed off-line using a program written in Matlab (Mathworks, UK)^9^.

Analysis of ventricular volumes, myocardial mass, ventricular function and extracellular volume fraction was performed using commercially available software cvi42 version 5.14.2 (Circle Cardiovascular Imaging, Canada) following a standard protocol taught by the software manufacturer. End-diastole was chosen as the point in the cardiac cycle when the blood pool is largest, and end-systole when the blood pool is smallest. The most basal short axis LV slice was defined as that in which 50% or more of the endocardial border consisted of ventricular myocardium. Endocardial and epicardial borders were outlined using computer assisted planimetry to obtain LV mass, end-diastolic and end-systolic volumes and LV ejection fraction. Papillary muscles were excluded from calculations of LV mass and were included as part of the myocardial blood pool. The subaortic LV outflow tract was included as part of the blood pool. RV endocardial borders were outlined at end-systole and end-diastole to calculate RV volumes and ejection fraction.

**Biomarkers**

The Roche Elecsys Troponin T hs assay has a limit of detection of 5 ng/L and a limit of quantification of 13 ng/L. The measuring range is 3-10,000 ng/L. The coefficient of variation (CV) for this assay is typically less than 10% at the 99th percentile, which is 14 ng/L for the general population.

The Roche Elecsys proBNP II assay has a measuring range of 10-35000 pg/mL (1.18-4130 pmol/L). Values below the limit of detection are reported as 10 pg/mL (1.18 pmol/L), and values above the measuring range are reported as > 35000 pg/mL (> 4130 pmol/L) or up to 70000 pg/mL (8260 pmol/L) for 2-fold diluted samples. The limit of quantitation is the lowest analyte concentration that can be reproducibly measured with an intermediate precision CV of ≤ 20%.

References for Supplement

1. Rauhalammi SMO, Mangion K, Barrientos PH, et al. Native myocardial longitudinal ( *T* _1_ ) relaxation time: Regional, age, and sex associations in the healthy adult heart. Journal of Magnetic Resonance Imaging. 2016 Sep;44(3):541–8.

2. Messroghli DR, Radjenovic A, Kozerke S, Higgins DM, Sivananthan MU, Ridgway JP. Modified Look-Locker inversion recovery (MOLLI) for high-resolutionT1 mapping of the heart. Magn Reson Med. 2004 Jul;52(1):141–6.

3. Giri S, Chung YC, Merchant A, et al. T2 quantification for improved detection of myocardial edema. Journal of Cardiovascular Magnetic Resonance. 2009 Dec 30;11(1):56.

4. Haaf P, Garg P, Messroghli DR, Broadbent DA, Greenwood JP, Plein S. Cardiac T1 Mapping and Extracellular Volume (ECV) in clinical practice: a comprehensive review. Journal of Cardiovascular Magnetic Resonance. 2017 Jan 30;18(1):89.

5. Mangion K, Clerfond G, McComb C, et al. Myocardial strain in healthy adults across a broad age range as revealed by cardiac magnetic resonance imaging at 1.5 and 3.0T: Associations of myocardial strain with myocardial region, age, and sex. Journal of Magnetic Resonance Imaging. 2016 Nov;44(5):1197–205.

6. Spottiswoode BS, Zhong X, Hess AT, et al. Tracking Myocardial Motion From Cine DENSE Images Using Spatiotemporal Phase Unwrapping and Temporal Fitting. IEEE Trans Med Imaging. 2007 Jan;26(1):15–30.

7. Spottiswoode BS, Zhong X, Lorenz CH, Mayosi BM, Meintjes EM, Epstein FH. Motion-guided segmentation for cine DENSE MRI. Med Image Anal. 2009 Feb;13(1):105–15.

8. Kellman P, Hansen MS, Nielles-Vallespin S, et al. Myocardial perfusion cardiovascular magnetic resonance: optimized dual sequence and reconstruction for quantification. Journal of Cardiovascular Magnetic Resonance. 2017 Apr 7;19(1).

9. Gilliam AD, Epstein FH. Automated Motion Estimation for 2-D Cine DENSE MRI. IEEE Trans Med Imaging. 2012 Sep;31(9):1669–81.
